# Specific Binding of the Pathogenic Prion Isoform: Development and Characterization of a Humanized Single-Chain Variable Antibody Fragment

**DOI:** 10.1371/journal.pone.0015783

**Published:** 2011-01-20

**Authors:** Nives Škrlj, Tanja Vranac, Mara Popović, Vladka Čurin Šerbec, Marko Dolinar

**Affiliations:** 1 Biochemistry Chair, Faculty of Chemistry and Chemical Technology, University of Ljubljana, Ljubljana, Slovenia; 2 Department for Production of Diagnostic Reagents and Research, Blood Transfusion Centre of Slovenia, Ljubljana, Slovenia; 3 Institute of Pathology, Faculty of Medicine, University of Ljubljana, Ljubljana, Slovenia; Ohio State University, United States of America

## Abstract

Murine monoclonal antibody V5B2 which specifically recognizes the pathogenic form of the prion protein represents a potentially valuable tool in diagnostics or therapy of prion diseases. As murine antibodies elicit immune response in human, only modified forms can be used for therapeutic applications. We humanized a single-chain V5B2 antibody using variable domain resurfacing approach guided by computer modelling. Design based on sequence alignments and computer modelling resulted in a humanized version bearing 13 mutations compared to initial murine scFv. The humanized scFv was expressed in a dedicated bacterial system and purified by metal-affinity chromatography. Unaltered binding affinity to the original antigen was demonstrated by ELISA and maintained binding specificity was proved by Western blotting and immunohistochemistry. Since monoclonal antibodies against prion protein can antagonize prion propagation, humanized scFv specific for the pathogenic form of the prion protein might become a potential therapeutic reagent.

## Introduction

For more than thirty years murine monoclonal antibodies have been routinely produced by the Köhler and Milstein method [1]. Such mAbs are widely used in clinical diagnostics, but are not appropriate for human therapy, since they elicit an immune response, referred to as human anti-mouse antibodies (HAMA) [2]. With the advances in molecular genetics and DNA technology less immunogenic recombinant antibodies with binding properties similar to murine Abs can be designed. The first attempt to minimize immunogenicity of murine antibodies was chimerization [3] where murine variable regions were fused to human constant regions. However, chimeric antibodies can still trigger HACA (human anti-chimeric antibodies) response. To further reduce the immunogenicity, CDR (complementarity determining regions) grafting was developed [4] in which hypervariable regions of a murine antibody are introduced into a human antibody using genetic manipulation. Although such antibodies were proved to be less immunogenic, they frequently exhibited reduced affinity compared to the parent murine antibody. As an alternative to CDR grafting, resurfacing was developed, where only surface residues of variable regions are replaced with those found in human antibodies [5]. It was based on the premise that the human immune response is directed mainly to the surface residues. With unchanged amino acids in the core of variable regions, conformation of the antigen binding site is less likely to be disturbed, thus the specificity and affinity of the parent antibody should be maintained.

In 1986 the first murine monoclonal antibody (mAb) was approved for clinical use by the Food and Drug Administration and since then more than 20 mAbs have been approved for therapeutic applications in humans. Humanized antibodies represent more than a half of them [6], [7].

Antibody-based immunotherapy might represent an effective treatment for several diseases [8] including conformational disorders like transmissible spongiform encephalopathies (TSEs) [9]. The hallmark of these diseases is the conformational change of the host-encoded cellular prion protein (PrP^C^) into the pathogenic isoform (PrP^Sc^), named prion [10]. Despite all the effort put into research of prion diseases, some basic mechanisms of the prion neurotoxicity and pathogenesis remain unclear. This is one of the reasons why neither therapy for TSE nor tools for an early *pre mortem* diagnostics of asymptomatic prion-infected individuals are available at the moment. Numerous compounds were tested for their antiprion activity [for review see 11] and the use of monoclonal antibodies seems to be one of the most promising therapeutic approaches. Since the first successful production of high-affinity anti-prion protein (PrP) antibodies in PrP-knockout mice [12], several mAbs against PrP have been produced. However, only a few mAbs capable to distinguish PrP^Sc^ form PrP^C^ have beed reported [13], [14], [15], [16], [17], [18]. One of them is mAb V5B2, prepared against the C-terminal peptide P1 of the human PrP [13]. Many reports have shown that some of murine anti-PrP mAbs that did not distinguish between PrP^C^ and PrP^Sc^ were nevertheless able to prevent prion infection *in vitro* [19], [20], [21], [22], and a few studies also demonstrated *in vivo* anti-prion action of such mAbs [9], [23], [24]. However, as PrP^C^ is normally expressed on the surface of a variety of cell types, doubts about possibly deleterious systemic blocking of PrP^C^ by such isoform-nonspecific mAbs have emerged [19], [25]. A range of antibody fragments with anti-prion activity has been derived from murine anti-PrP antibodies [26] and a few antibody fragments were already isolated from combinatorial phage display libraries expressing human scFvs [27], [28].

V5B2 is the PrP^Sc^-specific IgG1 monoclonal antibody prepared against a synthetic peptide P1, chosen from the C-terminus of the human PrP. It was shown that it distinguishes between brain tissue samples from CJD (Creutzfeldt–Jakob disease) - affected and non-CJD-affected patients reacting only with the native PrP^Sc^ deposits in immunohistochemical assays [13]. Because of these properties, it has a great potential to be used in immunodiagnostic procedures or prion research. It was already used to induce anti-idiotypic response in mice and chicken in order to produce anti-idiotypic antibodies, which represented a new experimental approach in prion research [29]. To better understand the narrow selectivity of V5B2, interaction between the antibody and P1 peptide was investigated [30], but only the most recent studies revealed that V5B2 selectively recognizes the newly discovered C-terminally truncated PrP, named PrP226* (S. Koren, personal commun.). To prepare a more suitable form of V5B2 for further immunotherapeutic development, recombinant single-chain antibody fragments have been derived from the mAb [31]. However, murine antibody fragments are immunogenic, which is the major obstacle to their clinical application. For that reason, we decided to reduce its immunogenicity by humanization.

The present data describe the humanization of the antibody single-chain fragment (scFv) V5B2 and its characterization. To our knowledge, this is the first report of an anti-PrP mAb being humanized. We rationally designed four variants of humanized scFvs V5B2 by resurfacing of variable regions guided by computer modelling. By site-directed mutagenesis, human amino acid residues were stepwise introduced into murine variable regions. After being produced in *E. coli* using pMD204 expression vector [32], humanized antibody fragments were purified from the periplasm and their antigen-binding properties were analysed. The optimized construct was a scFv with 13 mutations introduced at key positions in the structure, which retained stability, binding specificity and affinity of the parent antibody. We believe that the recombinant humanized scFv with preserved functional properties of V5B2 could be used for designing new compounds with potentially diagnostic and therapeutic anti-prion properties.

## Materials and Methods

### 1. Ethics statement

Approval for research involving human material has been obtained from the Slovenian National Medical Ethics Committee with decision dated January 15, 2008. *Post mortem* brain tissue of a patient who was clinically suspected for CJD was analyzed by immunohistochemistry without patient's consent because such analysis is obligatory by a ministerial decree in purpose of TSE surveillance (Official Gazzette of the Republic of Slovenia, 2/2001).

### 2. Reagents and strains

DNA restriction and modification enzymes were from Fermentas, except Pfx50 polymerase (Invitrogen) and Taq DNA ligase (New England BioLabs). Molecular weight standards and DNA purification kits were obtained from Fermentas as well. Oligonucleotides were from Invitrogen. For all cloning experiments we used *E. coli* strain DH5α. For expression, BL21[DE3] strain was used. Preparation of recombinant full length human prion protein HuPrP(23–231) and its fragment HuPrP(23–226), also named PrP226*, was performed in the laboratory of Dr. Giuseppe Legname (Italian Institute of Technology - SISSA, Trieste, Italy). Synthetic peptides derived from the human prion protein (P1, amino acids 214–226: CITQYERESQAYY; P1Q, amino acids 214–227: CITQYERESQAYYQ) were purchased from JPT Peptide Technologies. Mouse mAb 6H4, recognizing the sequence DYEDRYYRE (human PrP residues 144–152) was purchased from Prionics, Zurich, Switzerland. Sections of paraformaldehyde-fixed, paraffin-embedded human cerebellar tissue samples from a patient with sporadic CJD (sCJD) with kuru plaques and synaptic immunohistochemical type of prion deposition pattern were used in the study. Tissue samples for immunohistochemistry analyses were obtained from the Institute of Pathology, University of Ljubljana Medical Faculty.

### 3. Monoclonal antibody V5B2 and murine scFv V5B2

Preparation of a hybridoma cell line secreting a monoclonal IgG1 directed against the P1 peptide of human prion protein has been reported previously [13]. Total RNA was isolated from hybridoma cells as described [33]. Complete coding regions of both variable domains were obtained and single-chain antibody fragments were prepared in bacterial cells as described [31].

### 4. V5B2 framework analysis: Humanization design

**Figure 1:**
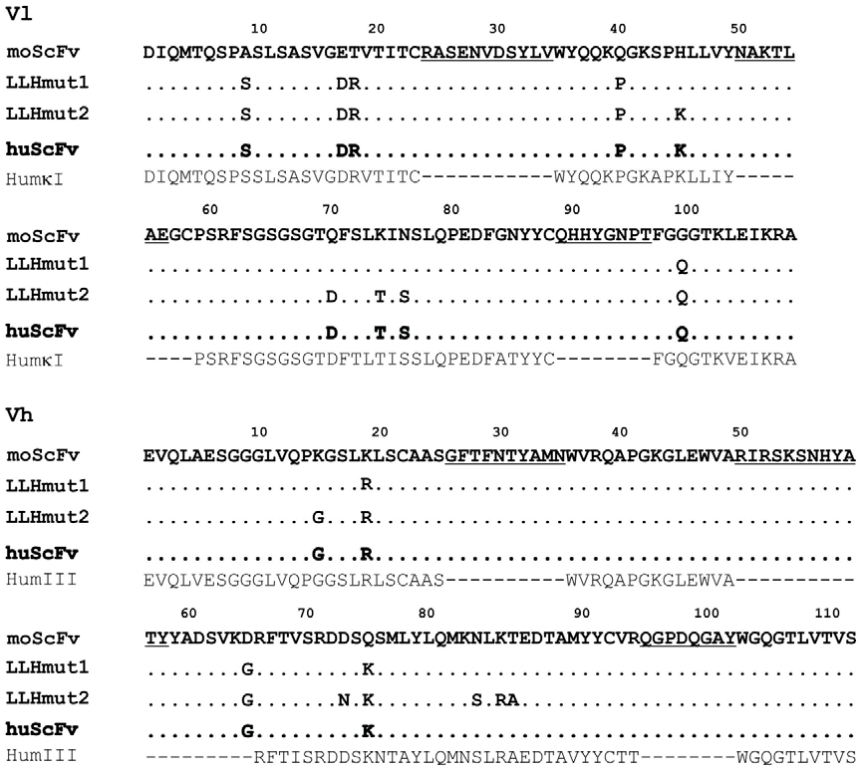
Amino acid sequence alignments. Alignment of light chain (Vl) and heavy chain (Vh) variable regions of the murine V5B2 scFv (moScFv), two humanized (LLHmut1, LLHmut2) and final humanized version (huScFv) of mAb V5B2 with human consensus sequences of light chain κ subgroup I (HhumκI) and heavy chain subgroup III (HumIII). The dashes represent unchanged amino acids. CDRs are underlined. Amino acids are numbered according to Kabat [36].

Immunogenicity of murine antibody was minimized by humanization through antibody variable domains resurfacing. On the basis of sequence alignments framework residues in the murine antibody that are rare in human antibodies were identified and replaced with those usually found in structurally related human antibodies. Ten murine and ten human antibody sequences with highest identity to V5B2 were chosen from the 3D structure database (Protein Data Bank) [34]. Then, surface residues in antibody variable (Fv) domains (light chain and heavy chain variable domains (Vl + Vh)) of these selected antibodies were determined by calculation of residue solvent accessibilities using software Swiss-PdbViewer [35]. Residues with relative accessibility greater than 30% were defined as surface amino acids. Sequences of variable domains of murine antibody V5B2 were aligned with sequences of selected antibodies of known structure to identify homologous surface positions. The first set of highly homologous surface framework residues was selected from human sequences and was compared to those in the murine V5B2 in order to humanize them. Second, a set of framework residues that appeared to be on the surface of several selected human antibodies was identified and human residues at those positions were chosen to be introduced in the V5B2. Third, some framework non-surface residues that appeared to be highly conserved in human antibodies but were different in V5B2 were candidates for replacement. Fourth, Ser43 in Vl of V5B2 located at the Vl/Vh interface differed from the human consensus residue for this position and was thus also chosen for replacement. We prepared a structure model of V5B2 Fv to analyse the influence of each mutation on V5B2 CDRs conformation. All variants of V5B2 were modelled and tested *in silico* for antigen binding. The humanized sequence of V5B2 variable domains was compared to human VH subgroups HhumκI and HumIII [36] sequences and the identity and similarity were calculated by ClustalW [37]. Detailed description of the stepwise humanization process is presented in Results and amino acid sequence alignment is shown in Figure 1.

### 5. Molecular modelling of V5B2 variable domains

**Figure 2:**
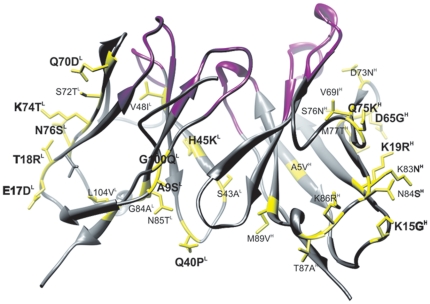
Molecular model of the Fv V5B2. CDR loop residues are coloured pink. Non-conserved surface residues are shown in yellow. Mutations introduced into the huScFv are bolded.

Molecular model of the murine V5B2 Fv was generated via homology-based modelling using a computer program Modeller9v1 [38]. Nine antibody structures with the highest sequence identity with the V5B2 were selected from the PDB database and used as templates to generate a model. Three of the selected structures were used as templates for Vl (identity 68–92%), five of them as templates for Vh (identity 66–86%) and one structure was used as a template for proper orientation of variable domains in Fv. First, ten basic models were build, followed by loop refinement for L3 and H3 loops, which resulted in a total of fifty models. The model with the lowest modeller objective function and optimal DOPE-score was selected as the final model. The quality of the model was analysed by a range of tools for evaluation of crystallographic models. V5B2 Fv model of is shown in Figure 2.

### 6. Site-directed mutagenesis

All mutations were introduced into V5B2 variable domains using the mutagenesis by incorporation of a phosphorylated oligonucleotide during polymerase chain reaction (PCR) amplification essentially as described [39]. Briefly, three primers (forward, reverse and mutagenic) were used to generate each mutant: first, the mutagenic oligonucleotide was phosphorylated using T4 polynucleotide kinase and added directly to the amplification reaction together with thermostable DNA ligase. After PCR, two products were observed. The full-length product was purified, ligated into the cloning vector pJET1/blunt (Fermentas) and sequenced (Macrogen).

### 7. Construction, expression and purification of humanized scFvs

Humanized scFvs were produced as described for murine scFvs V5B2 [31]. Mutated coding regions for heavy chain variable domain (Vh) and light chain variable domain (Vl) were amplified by PCR using specific primers. Vl and Vh were inserted into pMD204 expression vector between *Nru*I and *Eco*RI and *Xho*I and *Hin*dIII restriction sites, respectively. The 15-amino acid linker (Gly_4_Ser)_3_ was obtained from a pair of phosphorylated oligonucleotides and the cassette was used to connect the C-terminus of Vl to the N-terminus of Vh. Five final vectors, named pMD204-LLHmut1, pMD204-LLHmut2, pMD204-LLHmut3, pMD204-LLHmut4 and pMD204-LLHhum, were constructed. After transformation of *E. coli* DH5α, vector isolation and sequencing of the inserts, recombinant vectors were used for *E. coli* BL21[DE3] transformation. Transformants were grown in M9 minimal medium supplemented with 2% glucose, 0.5% peptone, and chloramphenicol (50 mg/ml) at 37°C and 250 rpm. Isopropyl β-D-1-thiogalactopyranoside (IPTG) was added to a final concentration of 0.5 mM as the OD550 of cell culture reached 1.0 and cell growth was continued for 12–16 h at 16°C and 200 rpm. Cells were harvested and resuspended in a small volume of 50 mM sodium phosphate, pH 8.0, 0.3 M NaCl, sonicated and pelleted by centrifugation at 15500 g for 30 min and 4°C. Supernatant was collected as soluble fraction. Cell lysates were prepared for electrophoresis as described [31].

Single-chain Fvs were purified by immobilized metal ion affinity chromatography (IMAC). The soluble fraction was loaded onto the column and washed with the washing buffer (50 mM sodium phosphate, pH 8.0, 0.3 M NaCl, 10 mM imidazole). The recombinant scFv was eluted with the elution buffer (50 mM sodium phosphate, pH 8.0, 0.3 M NaCl, 250 mM imidazole). Collected fractions were pooled, dialyzed against PBS and analysed on 15% polyacrylamide slab gels according to Laemmli [40] under reducing conditions. Anigen-binding properties of recombinant scFvs were analysed by enzyme-linked immunosorbent assay (ELISA), Western blotting and immunohistochemistry (IHC).

### 8. ELISA

Microtiter plates were coated overnight at 4°C with 50 µl of 5 µg/ml peptide P1 or P1Q in 50 mM carbonate/bicarbonate buffer pH 9.6. After blocking with 1% bovine serum albumin in 10 mM phosphate buffer (pH 7.2) and washing, purified scFvs (ten-fold serial dilutions, at concentrations ranging from 300 µg/ml to 300 pg/ml) were added and incubated for 1 h at 37°C. Single-chain Fvs were detected using primary mouse anti-His_6_ antibodies (Roche) and secondary goat anti-mouse IgG antibodies conjugated with horseradish peroxidase (HRP) (Jackson ImmunoResearch). Incubation was performed at 37°C for 1 h. Peroxidase activity was detected using the peroxidase substrate ABTS (2,2′-azino-bis(3-ethylbenzthiazoline-6-sulphonic acid) (Sigma). After 20-min incubation at 37°C, absorbance at 405 nm was measured. The absorbance signal at 405 nm versus the total concentration of scFv added to the wells was plotted. Experimental data were fitted to equation S = Smax*Ab/(Ab+K_D_).

### 9. Immunohistochemistry

Tissue samples were immersed in 96% formic acid for 1 h after fixing in paraformaldehyde. Sections were deparaffinized and pretreated for optimal antigen retrieval by 30 min autoclaving at 121°C in distilled water, followed by a 5 min incubation in 96% formic acid. Pretreated sections were blocked in 1% bovine serum albumin solution for 20 min at room temperature (RT). They were subsequently incubated overnight with primary antibodies (5 µl/ml V5B2 and 100 µg/ml mouse LLH scFv (moScFv) or humanized scFv (huScFv)) at RT in the moist chamber. Primary antibody concentrations were chosen from ELISA titration curves in order to obtain optimal antigen binding. Brain tissue sections incubated with scFvs were washed and incubated for 3 h with anti-His_6_ antibodies (0.3 ug/ml, Roche) at RT. Finally, all sections were washed and incubated for 1.5 h with anti-mouse HRP-labelled antibodies (diluted 1∶1000, Jackson ImmunoResearch) at RT. After thorough rinsing, sections were developed in 3,3′-diaminodbenzidine (DAB) chromogen for 5 min. Brain tissue counterstaining was obtained by 2 min immersion of sections in Mayer's hematoxylin.

### 10. SDS-PAGE and immunoblotting

Cell lysates were incubated for 5 min at 95°C in 5× SDS loading buffer containing 25% 2-mercaptoethanol. Samples were loaded on 12% SDS-Tris-glycine polyacrylamide gels and electrophoresed for 90 minutes at 130 V. Proteins were blotted onto 0.2 µm nitrocellulose membranes (Protran BA83, Whatman) at 210 mA for 70 min. Membranes were blocked with 5% non-fat milk at 4°C overnight, washed and then incubated with primary mAb V5B2 (5 µg/ml in 1% non-fat milk solution), 6H4 (0.2 µg/ml in 1% non-fat milk solution), moScFv or huScFv (35 ug/ml 1% non-fat milk solution) for 90 minutes with shaking at RT. After washing, membranes incubated with scFvs were incubated with anti-His_6_ antibodies (0.3 µg/ml, Roche) for 90 min with shaking at RT, then with HRP-labelled anti-mouse secondary antibody (Jackson ImmunoResearch; 1∶5000 in 1% skimmed milk solution) at room temperature for 90 minutes, and washed again. Reaction was detected using Amersham ECL Western blotting detection reagents.

## Results

### 1. Humanization guided by computer modelling

Humanization by antibody variable domain resurfacing was chosen for minimization of immunogenicity of the murine antibody V5B2. Murine residues that should be replaced were determined based on the sequence alignment of V5B2 with other murine and human sequences with the identities ranging from 87% to 64% for Vh and 92% to 69% for Vl. Surface-accessible residues of Fv regions were defined as those with relative solvent accessibility ≥30%. Fifteen such residues in Vl and thirteen residues in Vh were determined on the surface of the V5B2 framework, which was consistent with results obtained by statistical analysis of a database of murine and human immunoglobulin sequences described in the Pedersen study [41]. Seven of these residues (4 in Vl and 3 in Vh) were non-conserved and were chosen to be replaced with the human consensus residues for these positions. In addition, V5B2 model Fv (Fig. 2) revealed that L17 residue might be considered as surface accessible, therefore together eight replacements were made in the first version of humanized V5B2 scFv, named LLHmut1. With further investigation, additional 14 residues were identified on the surface of each variable region of several structurally related human antibodies. Analysis on the V5B2 model revealed that replacement of 4 non-conserved residues in Vl and 5 non-conserved residues in Vh should not significantly alter the CDRs conformation. These 9 replacements were thus additionally introduced in the second version of scFv V5B2, named LLHmut2. The basic goal of the resurfacing is replacement of only surface murine residues, retaining the core of variable regions as undisturbed as possible to avoid possible conformational perturbances of the CDRs. Since our aim was to make the murine antibody V5B2 as human-like as possible, we analysed V5B2 sequence further. Additional highly conserved residues were identified in the core of structurally related human antibody variable regions. Eleven residues in Fv V5B2 (5 in the core of Vl and 6 in the Vh) differed from human conserved amino acids at these positions. The introduction of human residues at selected positions in the murine model of V5B2 has not revealed major disturbances in the conformation of CDRs, so the third version of humanized scFv V5B2, LLHmut3, was designed. Finally, the influence of Ser43 at the Vl/Vh interface between murine and human antibody sequences was investigated. Since Ala is conserved residue for L43 position in human antibodies, murine Ser was replaced by Ala in the fourth version of humanized V5B2, named LLHmut4. All four humanized versions of V5B2 were prepared as recombinant proteins and analysed for antigen binding. Since LLHhum2, LLHmut3 and LLHmut4 all showed altered binding to P1, intermediate variants between LLHmut1 and LLHmut2 were prepared in the next round of humanization. The final humanized V5B2, named huScFv, contained 9 mutations in Vl and 4 mutations in Vh and had binding properties similar to mouse scFv directed against the same antigen. In order to preserve strong binding activity, 6 murine residues in Vl and 9 in Vh were maintained in the frameworks. Sequences of murine and humanized V5B2 variable regions aligned with human consensus sequences of heavy chain subgroup III (HumIII) and light chain κ subgroup I (HhumκI) are shown in Figure 1. Based on amino acid sequence alignment, identity and similarity scores of the V5B2 humanized variable domain frameworks with HumκI and HumIII human antibody frameworks [36] were calculated by ClustalW. *In silico* analysis showed that amino acid sequence of huScFv appeared to be highly similar to amino acid sequence of human antibodies with most of the differences in the FR3 region of the Vh domain. The results are shown in Table 1.

**Table 1:** Identity and similarity scores of the V5B2 humanized variable domain frameworks with HhumκI and HumIII human antibody frameworks calculated by ClustalW.

Domain	Identity (*X*/*Y*)	Identity (%)	Similarity (%)
**Vh**			
FR1	22/23	95.65	100
FR2	14/14	100	100
FR3	20/32	62.50	90.63
FR4	10/10	100	100
**Vl**			
FR1	23/23	100	100
FR2	13/15	86.67	100
FR3	27/30	90	90
FR4	11/12	91.66	100

*X* is the number of identical framework residues; *Y* is the total number of framework residues.

### 2. Production of humanized scFvs

**Figure 3:**
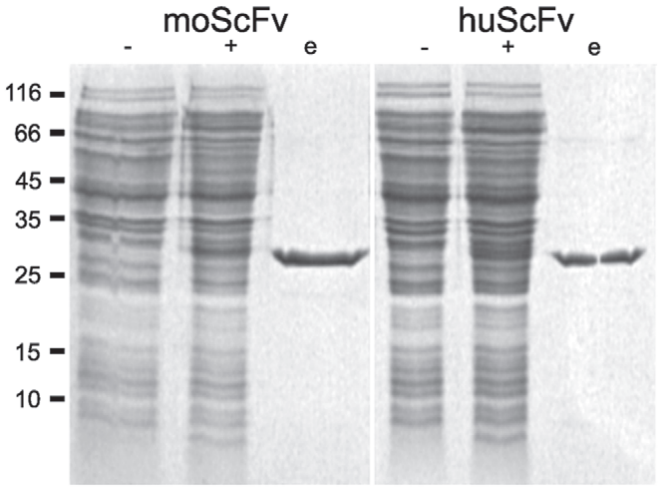
SDS-PAGE analyses of mouse (moScFv) and humanized (huScFv) scFv V5B2. Cell lysates were analyzed before (−) and after (+) IPTG induction, along with purified scFvs (e). Molecular masses of protein standards in kDa are indicated on the left. Similar electrophoretic pattern was obtained with all humanized scFv variants.

Complementary DNA encoding for V5B2 variable domains was derived from hybridoma cells mRNA and both chain orientations of murine scFvs V5B2 have been produced [31]. LLH chain orientation (scFv in Vl-linker-Vh chain arrangement) retained strong binding to the antigen [31], therefore the same orientation was chosen for the humanized V5B2 scFvs. All humanized scFvs were expressed in *E. coli* periplasm using the expression vector pMD204, specially developed for construction and production of fusion proteins [32]. The scFv-encoding constructs derived from variable regions of V5B2 linked via a short linker (G_4_S)_3_ were inserted into the pMD204 expression vector, in frame with the *omp*A signal sequence and upstream of the His10 tag. *E. coli* BL21[DE3] cells containing pMD204-scFv plasmid were induced by IPTG and then grown at 16°C for up to 18 h, since lowering incubation temperature improved the ratio of soluble to insoluble recombinant antibody fragments as already shown for murine scFvs [31]. The yield of recombinant proteins was similar for all humanized variants and was comparable to the production of murine scFv in LLH orientation. SDS-PAGE analyses of cell lysates revealed the presence of a band with the approximate molecular weight of 30,000 after induction, i.e. as expected for the scFv (Figure 3). No significant differences were observed in cell growth, protein production, purification or electrophoretic mobility between murine and humanized scFvs.

Nickel affinity chromatography was used for protein purifications, since His10 tag was added to the C-terminus of all recombinant scFvs. Most of contaminating proteins were eliminated from the soluble fraction of cell lysates by washing the column with a buffer containing 10 mM imidazole. Single-chain Fvs were eluted from the column with a buffer containing 250 mM imidazole. The final yield of purified scFvs was estimated at 0.5–1 mg per litre bacterial culture with purity greater than 90%. Purified scFvs were first analysed by SDS-PAGE (Fig. 3), and then tested for antigen binding by ELISA, Western blotting and IHC.

### 3. Antigen-binding properties

**Figure 4:**
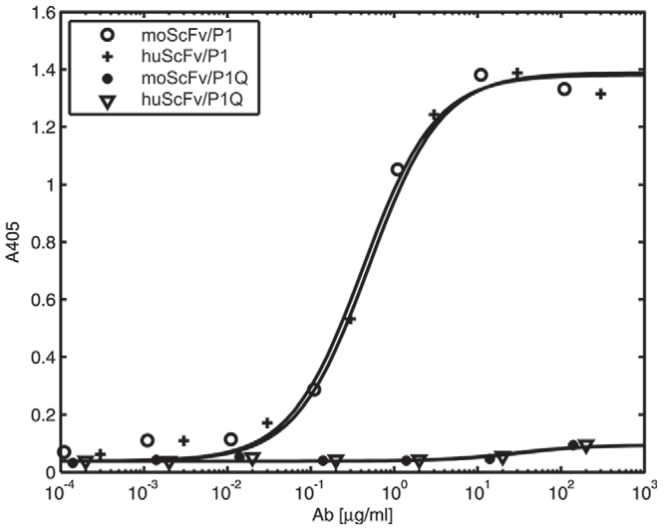
ELISA analysis of the antigen-binding efficiency of moScFv and huScFv. The absorbance at 405 nm is plotted against concentration of antibody fragment added to the wells coated with the P1 or P1Q peptide. The apparent affinity of moScFv in comparison with Fab V5B2 has been discussed previously [31].

The ability of humanized variants of scFv V5B2 to bind peptide P1 was tested by ELISA (Figure 4). LLHmut1 showed similar binding to P1 peptide as moScFv, while other three humanized variants successfully bound P1 only when ELISA was performed at lower temperature (e.g. 16°C) (data not shown). Since diagnostic and therapeutic reagent should retain their stability and activity at physiological temperatures, a variant between LLHmut1 and LLHmut2 with adequate binding properties was prepared. Binding affinity of the final humanized single-chain antibody fragment (V5B2 huScFv) was comparable to the affinity of moScFv V5B2. In addition, both scFvs, moScFv and huScFv, recognized the peptide P1, whereas no binding was observed when peptide P1Q (P1 prolonged for a single Glu residue at the C-terminus) was used as an antigen in ELISA (Figure 4). Similar binding specificity was shown for the parent antibody V5B2 [13].

**Figure 5:**
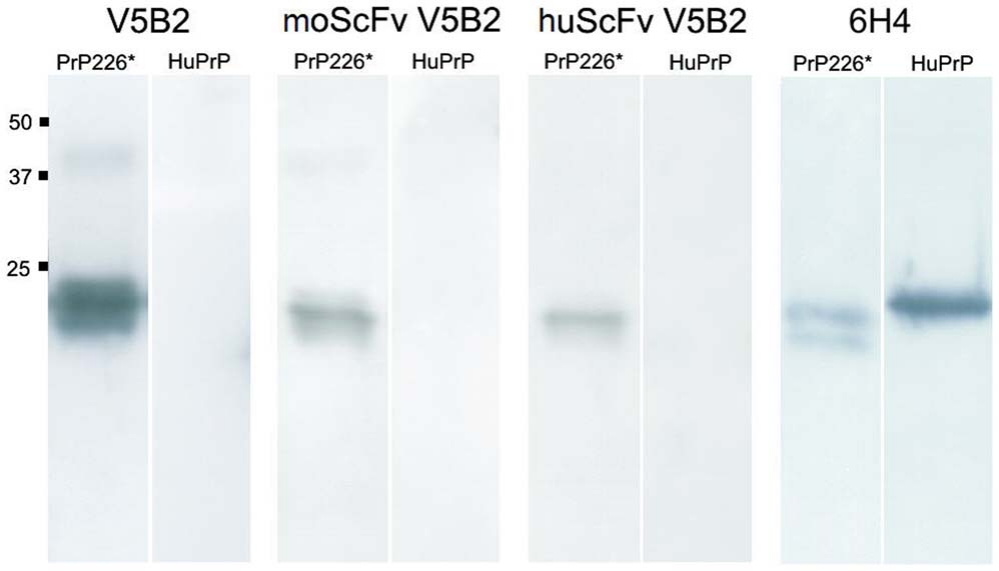
Western blotting of the recombinant human PrP 23–226 (PrP226*) and of the recombinant human PrP23–231 (HuPrP). Reaction with the whole mAb V5B2 is compared to reactions with murine scFv (moScFv) and humanized scFv (huScFv) of V5B2. mAb 6H4 was used as a control antibody. Approximate molecular weights are in kilodaltons.

**Figure 6:**
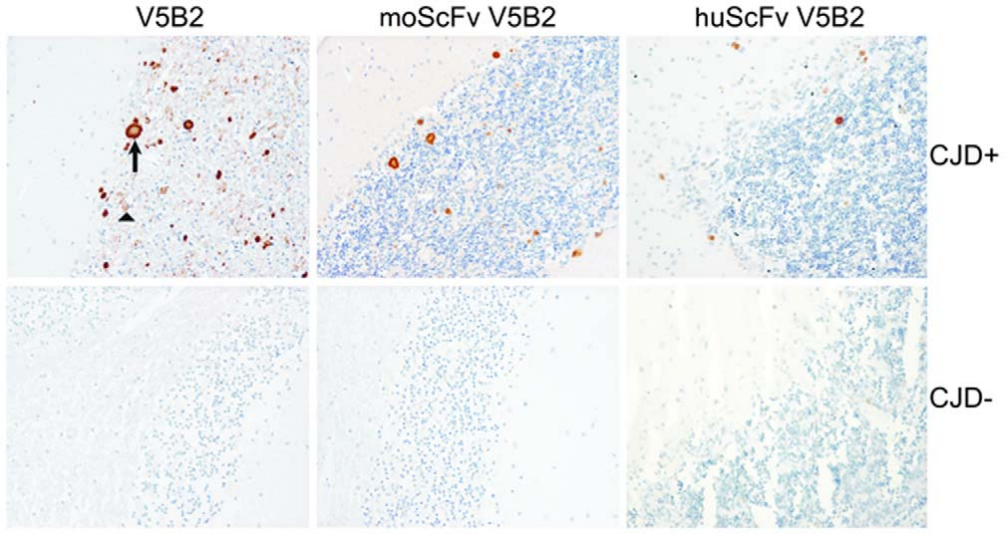
Immunohistochemistry of the PrP^Sc^ deposits in the cerebellum of a sCJD patient (upper three figures). Immunolabeling was performed with whole mAb V5B2, murine scFv (moScFv) and humanized scFv (huScFv) of V5B2. The arrow marks PrP^Sc^ plaques, while the triangle marks the diffuse, synaptic PrP^Sc^ deposition. On the lower three figures immunolabeling was performed on the cerebellum of the CJD negative patient.

Further, we demonstrated by immunoblotting that both murine and humanized scFvs efficiently labelled recombinant human PrP23–226, named PrP226* (S. Koren, personal commun.), but not the full-length PrP, and therefore retained the characteristic binding properties of V5B2 (Figure 5). The results were compared to mAb 6H4 binding, which does not distinguish between cellular and pathological isform of PrP [16]. In contrast to V5B2 antibody forms, it labelled PrP226* as well as full-length PrP (Figure 5). Finally, we performed IHC on a tissue sample of a sCJD case with kuru plaque and synaptic pattern of PrP^Sc^ deposition with moScFv, huScFv or whole V5B2 antibodies. The results show that both scFvs specifically label kuru plaque PrP^Sc^ deposits, but were unable to label the fine synaptic PrP^Sc^ deposition pattern, both finely labelled with V5B2 antibody (Figure 6).

## Discussion

Single-chain Fvs are much smaller than whole antibodies, besides they do not require glycosylation and can be produced in a bacterial expression system. Such production is simpler, faster and significantly reduces production costs [42]. Therefore, instead of full-length V5B2 antibody the scFv form was chosen to be humanized and produced.

We have successfully humanized a single-chain antibody fragment of the murine monoclonal antibody V5B2, specific for PrP^Sc^-fragment associated with prion-infected samples [13]. We constructed over 30 scFvs on the DNA level and expressed, purified and examined a total of 14 humanized constructs: 4 for basic stages and 10 intermediates between different stages in search of destabilizing mutations. Construct with optimal binding properties and maximal possible amino acid replacements was denoted huScFv V5B2. Our humanization approach by resurfacing differed from the conventional one, as beside surface residues we tried to replace some residues in the core of variable regions as well. Non-conserved framework surface residues, which were not deemed too close to CDRs were replaced, but experimental analyses revealed that of these, 6 positions in Vl and 10 positions in Vh substantially affected antibody interaction with the antigen. For that reason a compromise between potential immunogenicity and retained binding specificity had to be made. Finally, 13 murine residues were replaced in the moScFv V5B2 to prepare a huScFv V5B2, which retained the significant ability to discriminate between CJD-affected and normal brain tissue. Nevertheless, the amino acid sequence of huScFv V5B2 shows high similarity with the human heavy chain subgroup III and light chain κ subgroup I consensus sequences.

In the process of humanization a computer model of antibody variable domains is often built to help design the humanized form. It is used for prediction of possible influence of mutations on CDR conformations, which usually results in loss of antibody binding affinity or even specificity. Several reports showed that analysis of a computer model actually helped to avoid problems with the affinity reduction, which is particularly typical for CD-grafting [43], [44], [45]. Moreover, it was demonstrated that during humanization antibody affinity could even be improved when the humanized form is carefully designed on the basis of a precise analysis of structural models [46]. In our case, the structural model of the Fv V5B2 helped to determine framework surface residues of the V5B2, but it did not predict negative impact of several replacements we have introduced. It was however reasonable to expect that not all planned mutations could be introduced into variable domains without disturbing the structure of the antigen-binding site and influencing the binding. For that reason, several intermediate variants were prepared and tested for antigen-binding activity.

A few resurfaced scFvs have been reported in the literature, generally containing from six to ten replacements [e.g. 45], [47], [48]. Any additional mutation usually resulted in reduced binding activity. It was also shown that even a single mutation in the antibody framework can improve or reduce the expression yield or binding affinity of a scFv tremendously [49], [50], [51].

In our experiments, Western blot analyses indicated that humanized V5B2 scFv recognized the same epitope on PrP as the parent V5B2 mAb. When huScFv was assayed by IHC, it labelled less kuru plaque-like PrP^Sc^ deposits than V5B2 and failed to label the synaptic pattern of PrP^Sc^ deposition, which was clearly visualized by whole V5B2 mAb. This observation was attributed to expected reduced affinity of scFvs that hindered the detection of fine synaptic pattern and small plaques. Our IHC experiment clearly demonstrated that both murine and humanized form of scFv retained the ability to label PrP^Sc^ deposits specifically, although less potently, which is in agreement with results obtained by ELISA and immunoblotting.

Even though several antibodies have been resurfaced in the last decade, their immunogenicity remains undetermined, since no clinical data on resurfaced antibodies has been published yet [52]. Since the amino acid sequence of the humanized scFv was carefully designed and was found to be highly similar to human sequences, we believe that we efficiently removed all major immunogenic epitopes on the murine antibody. However, the actual immunogenicity could only be determined in clinical trials.

Immunotherapy based on anti-PrP antibodies is a promising strategy for the treatment of prion diseases. It has already been shown that some anti-PrP mAbs can antagonize prion propagation *in vitro* and *in vivo*, but only outside the brain, most likely due to very limited entry of large molecules into the central nervous system.

Single-chain fragments are much smaller than whole antibodies, but usually they retain specific monovalent antigen-binding affinity of the parent antibody, with improved pharmacokinetics for tissue penetration. Antibody fragments have already been reported to be successfully delivered to the central nervous system by intranasal administration [53], by virus-mediated gene transfer system [54] or by re-engineering as fusion proteins with BBB molecular Trojan horses [55]. Besides, antibody fragments appear to be more appropriate for TSE treatment than full antibodies, since bivalent anti-PrP antibodies have been shown to cross-link PrP^C^ molecules and trigger neuronal apoptosis in certain neuronal populations [56]. It was also demonstrated that constant domains are unnecessary for antiprion effect, since Fab D18 [22], scFv 6H4 [57] and scFv D18 [58] all exhibited antiprion activity. A construct that targets PrP^Sc^ specifically could even be more efficient.

Moreover, distinguishing between the pathological and the normal isoform of PrP is one of the most desirable properties of diagnostic tools for prion diseases. Based on the existing knowledge it can be concluded that small molecules, exhibiting high affinity binding of the pathological PrP isoform, such as our humanized scFv V5B2, might be a potential therapeutic reagent for TSEs.

## References

[1] Kohler G, Milstein C (1975). Continuous cultures of fused cells secreting antibody of predefined specificity.. Nature.

[2] Mirick GR, Bradt BM, Denardo SJ, Denardo GL (2004). A review of human anti-globulin antibody (HAGA, HAMA, HACA, HAHA) responses to monoclonal antibodies. Not four letter words.. Q J Nucl Med Mol Imaging.

[3] Morrison SL, Johnson MJ, Herzenberg LA, Oi VT (1984). Chimeric human antibody molecules: mouse antigen-binding domains with human constant region domains.. Proc Natl Acad Sci U S A.

[4] Jones PT, Dear PH, Foote J, Neuberger MS, Winter G (1986). Replacing the complementarity-determining regions in a human antibody with those from a mouse.. Nature.

[5] Padlan EA (1991). A possible procedure for reducing the immunogenicity of antibody variable domains while preserving their ligand-binding properties.. Mol Immunol.

[6] Lefranc MP, Giudicelli V, Ginestoux C, Jabado-Michaloud J, Folch G (2009). IMGT, the international ImMunoGeneTics information system.. Nucleic Acids Res.

[7] Reichert JM, Rosensweig CJ, Faden LB, Dewitz MC (2005). Monoclonal antibody successes in the clinic.. Nat Biotechnol.

[8] Waldmann TA (2003). Immunotherapy: past, present and future.. Nat Med.

[9] White AR, Enever P, Tayebi M, Mushens R, Linehan J (2003). Monoclonal antibodies inhibit prion replication and delay the development of prion disease.. Nature.

[10] Prusiner SB (1982). Novel proteinaceous infectious particles cause scrapie.. Science.

[11] Ludewigs H, Zuber C, Vana K, Nikles D, Zerr I (2007). Therapeutic approaches for prion disorders.. Expert Rev Anti Infect Ther.

[12] Prusiner SB, Groth D, Serban A, Koehler R, Foster D (1993). Ablation of the prion protein (PrP) gene in mice prevents scrapie and facilitates production of anti-PrP antibodies.. Proc Natl Acad Sci U S A.

[13] Čurin Šerbec V, Bresjanac M, Popović M, Pretnar Hartman K, Galvani V (2004). Monoclonal antibody against a peptide of human prion protein discriminates between Creutzfeldt-Jacob's disease-affected and normal brain tissue.. J Biol Chem.

[14] Horiuchi M, Karino A, Furuoka H, Ishiguro N, Kimura K (2009). Generation of monoclonal antibody that distinguishes PrPSc from PrPC and neutralizes prion infectivity.. Virology.

[15] Jones M, Wight D, McLoughlin V, Norrby K, Ironside JW (2009). An antibody to the aggregated synthetic prion protein peptide (PrP106–126) selectively recognizes disease-associated prion protein (PrP) from human brain specimens.. Brain Pathol.

[16] Korth C, Stierli B, Streit P, Moser M, Schaller O (1997). Prion (PrP^Sc^)-specific epitope defined by a monoclonal antibody.. Nature.

[17] Paramithiotis E, Pinard M, Lawton T, LaBoissiere S, Leathers VL (2003). A prion protein epitope selective for the pathologically misfolded conformation.. Nat Med.

[18] Vranac T, Hartman KP, Popović M, Venturini A, Zerovnik E (2006). A single prion protein peptide can elicit a panel of isoform specific monoclonal antibodies.. Peptides.

[19] Beringue V, Vilette D, Mallinson G, Archer F, Kaisar M (2004). PrPSc binding antibodies are potent inhibitors of prion replication in cell lines.. J Biol Chem.

[20] Enari M, Flechsig E, Weissmann C (2001). Scrapie prion protein accumulation by scrapie-infected neuroblastoma cells abrogated by exposure to a prion protein antibody.. Proc Natl Acad Sci U S A.

[21] Féraudet C, Morel N, Simon S, Volland H, Frobert Y (2005). Screening of 145 anti-PrP monoclonal antibodies for their capacity to inhibit PrPSc replication in infected cells.. J Biol Chem.

[22] Peretz D, Williamson RA, Kaneko K, Vergara J, Leclerc E (2001). Antibodies inhibit prion propagation and clear cell cultures of prion infectivity.. Nature.

[23] Sadowski MJ, Pankiewicz J, Prelli F, Scholtzova H, Spinner DS (2009). Anti-PrP Mab 6D11 suppresses PrP(Sc) replication in prion infected myeloid precursor line FDC-P1/22L and in the lymphoreticular system in vivo.. Neurobiol Dis.

[24] Sigurdsson EM, Sy MS, Li R, Scholtzova H, Kascsak RJ (2003). Anti-prion antibodies for prophylaxis following prion exposure in mice.. Neurosci Lett.

[25] White AR, Hawke SH (2003). Immunotherapy as a therapeutic treatment for neurodegenerative disorders.. J Neurochem.

[26] Alexandrenne C, Hanoux V, Dkhissi F, Boquet D, Couraud JY (2009). Curative properties of antibodies against prion protein: a comparative in vitro study of monovalent fragments and divalent antibodies.. J Neurochem.

[27] Flego M, Ascione A, Zamboni S, Dupuis ML, Imperiale V (2007). Generation of human scFvs antibodies recognizing a prion protein epitope expressed on the surface of human lymphoblastoid cells.. BMC Biotechnol.

[28] Leclerc E, Liemann S, Wildegger G, Vetter SW, Nilsson F (2000). Selection and characterization of single chain Fv fragments against murine recombinant prion protein from a synthetic human antibody phage display library.. Hum Antibodies.

[29] Colja Venturini A, Bresjanac M, Vranac T, Koren S, Narat M (2009). Anti-idiotypic antibodies: a new approach in prion research.. BMC Immunol.

[30] Ulrih NP, Skrt M, Veranic P, Galvani V, Vranac T (2006). Oligomeric forms of peptide fragment PrP(214–226) in solution are preferentially recognized by PrP(Sc)-specific antibody.. Biochem Biophys Res Commun.

[31] Škrlj N, Čurin Šerbec V, Dolinar M (2010). Single-chain Fv antibody fragments retain binding properties of the monoclonal antibody raised against Peptide P1 of the human prion protein.. Appl Biochem Biotechnol.

[32] Škrlj N, Erčulj N, Dolinar M (2009). A versatile bacterial expression vector based on the synthetic biology plasmid pSB1.. Protein Expr Purif.

[33] Koren S, Kosmač M, Colja Venturini A, Montanič S, Čurin Šerbec V (2008). Antibody variable-region sequencing as a method for hybridoma cell-line authentication.. Appl Environ Microbiol.

[34] Berman HM, Westbrook J, Feng Z, Gilliland G, Bhat TN (2000). The Protein Data Bank.. Nucleic Acids Res.

[35] Guex N, Peitsch MC (1997). SWISS-MODEL and the Swiss-PdbViewer: an environment for comparative protein modeling.. Electrophoresis.

[36] Kabat EA, Wu TT, Perry HM, Gottesman KS, Foeller C (1991). Sequences of Proteins of Immunologcial Interest.

[37] Chenna R, Sugawara H, Koike T, Lopez R, Gibson TJ (2003). Multiple sequence alignment with the Clustal series of programs.. Nucleic Acids Res.

[38] Šali A, Blundell TL (1993). Comparative protein modelling by satisfaction of spatial restraints.. J Mol Biol.

[39] Michael SF (1994). Mutagenesis by incorporation of a phosphorylated oligo during PCR amplification.. Biotechniques.

[40] Laemmli UK (1970). Cleavage of structural proteins during the assembly of the head of bacteriophage T4.. Nature.

[41] Pedersen JT, Henry AH, Searle SJ, Guild BC, Roguska M (1994). Comparison of surface accessible residues in human and murine immunoglobulin Fv domains. Implication for humanization of murine antibodies.. J Mol Biol.

[42] Verma R, Boleti E, George AJ (1998). Antibody engineering: comparison of bacterial, yeast, insect and mammalian expression systems.. J Immunol Methods.

[43] Li B, Wang H, Zhang D, Qian W, Hou S (2007). Construction and characterization of a high-affinity humanized SM5-1 monoclonal antibody.. Biochem Biophys Res Commun.

[44] Queen C, Schneider WP, Selick HE, Payne PW, Landolfi NF (1989). A humanized antibody that binds to the interleukin 2 receptor.. Proc Natl Acad Sci U S A.

[45] Zhang W, Feng J, Li Y, Guo N, Shen B (2005). Humanization of an anti-human TNF-alpha antibody by variable region resurfacing with the aid of molecular modeling.. Mol Immunol.

[46] Luo GX, Kohlstaedt LA, Charles CH, Gorfain E, Morantte I (2003). Humanization of an anti-ICAM-1 antibody with over 50-fold affinity and functional improvement.. J Immunol Methods.

[47] Muzard J, Bouabdelli M, Zahid M, Ollivier V, Lacapere JJ (2009). Design and humanization of a murine scFv that blocks human platelet glycoprotein VI in vitro.. FEBS J.

[48] Delagrave S, Catalan J, Sweet C, Drabik G, Henry A (1999). Effects of humanization by variable domain resurfacing on the antiviral activity of a single-chain antibody against respiratory syncytial virus.. Protein Eng.

[49] Caldas C, Coelho V, Kalil J, Moro AM, Maranhão AQ (2003). Humanization of the anti-CD18 antibody 6.7: an unexpected effect of a framework residue in binding to antigen.. Mol Immunol.

[50] Knappik A, Pluckthun A (1995). Engineered turns of a recombinant antibody improve its in vivo folding.. Protein Eng.

[51] Roguska MA, Pedersen JT, Henry AH, Searle SM, Roja CM (1996). A comparison of two murine monoclonal antibodies humanized by CDR-grafting and variable domain resurfacing.. Protein Eng.

[52] Almagro JC, Fransson J (2008). Humanization of antibodies.. Front Biosci.

[53] Furrer E, Hulmann V, Urech DM (2009). Intranasal delivery of ESBA105, a TNF-alpha-inhibitory scFv antibody fragment to the brain.. J Neurochem.

[54] Wuertzer CA, Sullivan MA, Qiu X, Federoff HJ (2008). CNS delivery of vectored prion-specific single-chain antibodies delays disease onset.. Mol Ther.

[55] Boado RJ, Zhou QH, Lu JZ, Hui EK, Pardridge WM (2010). Pharmacokinetics and brain uptake of a genetically engineered bifunctional fusion antibody targeting the mouse transferrin receptor.. Mol Pharm.

[56] Solforosi L, Criado JR, McGavern DB, Wirz S, Sánchez-Alavez M (2004). Cross-linking cellular prion protein triggers neuronal apoptosis in vivo.. Science.

[57] Donofrio G, Heppner FL, Polymenidou M, Musahl C, Aguzzi A (2005). Paracrine inhibition of prion propagation by anti-PrP single-chain Fv miniantibodies.. J Virol.

[58] Campana V, Zentilin L, Mirabile I, Kranjc A, Casanova P (2009). Development of antibody fragments for immunotherapy of prion diseases.. Biochem J.

